# Beyond Withdrawal: Metabolic and Structural Causes of Seizures in Adults With Alcohol Use Presenting to the Emergency Department

**DOI:** 10.7759/cureus.95164

**Published:** 2025-10-22

**Authors:** Parv Modi

**Affiliations:** 1 Internal Medicine, Kasturba Hospital - Valsad, Valsad, IND

**Keywords:** alcohol dependence, alcohol misuse, alcohol withdrawal, clinical triage, electrolyte imbalance, emergency department, hypoglycaemia, ketoacidosis, seizures

## Abstract

Background

In adults with a history of alcohol use who present with a seizure, alcohol withdrawal is often presumed, but alternative and immediately treatable causes are common. We tried to quantify the proportion of alcohol-withdrawal-related seizures versus other causes, and to identify bedside features that help differentiate them.

Methods

We conducted a single-centre observational cohort study of adults presenting to the Emergency Department with a seizure and any history of alcohol use. Cases were classified as alcohol-withdrawal-related or due to other causes using prespecified clinical criteria that incorporated hours since last drink; a structured withdrawal assessment (Clinical Institute Withdrawal Assessment for Alcohol-Revised, or CIWA-Ar); laboratory data (glucose, ketones or anion-gap acidosis, electrolytes including magnesium); neuroimaging, whenever performed; and clinical documentation. We described presentation characteristics, laboratory profiles, and in-hospital course, including ICU care, seizure recurrence, length of stay, and death.

Results

Among 500 participants, 190 (38.0%) were alcohol-withdrawal-related, and 310 (62.0%) were due to other causes. Within the non-withdrawal group, proximate causes were metabolic derangements with ketoacidosis (80, 16.0%), low blood sugar (70, 14.0%), primary electrolyte disturbance (50, 10.0%), structural brain disease (45, 9.0%), and other or combined causes (65, 13.0%). Timing strongly discriminated groups: withdrawal cases clustered at 6-48 hours after the last drink (46.3% at 6-24 hours; 36.8% at 24-48 hours), whereas other causes were more dispersed in time, including 17.7% beyond 72 hours. At triage, the median withdrawal score was higher in the withdrawal group (CIWA-Ar: 18 vs. 6), with more frequent seizure clustering. Metabolic abnormalities were substantially more common in other causes (low blood sugar, ketones or anion-gap acidosis, low blood sodium, low blood potassium, low blood magnesium). Overall, 12.0% required ICU/HDU (High Dependency Unit) care, 11.0% had in-hospital seizure recurrence, the median length of stay was four days (interquartile range, 2-6), and in-hospital death occurred in 2.4%.

Conclusions

Most seizures in adults with alcohol use were not due to alcohol withdrawal. A simple triage approach - time since last drink, structured withdrawal assessment, and rapid bedside testing for glucose and electrolytes - reliably separates withdrawal from other causes and directs early, reversible treatment.

## Introduction

Alcohol harms are significant, and much of it is preventable. Classic bedside descriptions still guide how we recognize alcohol use disorders, while modern public health data show just how large the problem is in India and worldwide [1-4]. Using a shared diagnostic language helps teams align their decisions in the Emergency Department (ED) and on the ward, separating harmful use, dependence, and withdrawal [5,6].

In real life, we often meet a patient with a first seizure, and when there’s alcohol in the story, it’s easy to call it an alcohol-withdrawal seizure (AWS). But alcohol and seizures cross paths in a few different ways: long-term drinking lowers the seizure threshold, can sit alongside structural brain disease, and often comes with metabolic problems that trigger seizures on their own [7-13]. AWS has a familiar tempo, i.e., mostly generalized tonic-clonic seizures (GTCS) appear 6-48 hours after the last drink, and episodes may get worse with repeated withdrawals known as kindling, which matches what we know about the brain shifting toward less gamma-aminobutyric acid (GABA) inhibition and more glutamate excitation [7,10,12-15].

Because not every seizure in a drinker is withdrawal-driven, the first hour really matters. Take a focused history (especially when they last drank) and score withdrawal properly (Clinical Institute Withdrawal Assessment for Alcohol-Revised, or CIWA-Ar); check glucose early, look at acid-base balance/ketones and routine electrolytes; and use neuroimaging when the story is focal, atypical, or just doesn’t add up. Treatment should follow risk-stratified pathways, with benzodiazepines being the first-line drugs when AWS is likely, keeping people safe without over- or undertreating [16-20].

Don’t miss the mimics. In people who drink, alcoholic ketoacidosis (AKA) and hypoglycaemia show up a lot, and they’re time-critical. Reports, old and new, describe the typical pattern (anion-gap metabolic acidosis, ketonaemia after poor intake or vomiting), how to spot it in the ED, and what to do: glucose, fluids, thiamine before carbs, and correct electrolytes [11,19,21-24]. Given the overlap with malnutrition and thiamine deficiency, keep a low threshold for early parenteral thiamine to prevent Wernicke-Korsakoff syndrome [24].

Indian patient demographics isn’t one thing either. Drinking patterns, access to emergency care, and seizure phenotypes vary quite a bit, so centres shouldn’t assume every seizure in a drinker is AWS. Local profiling helps teams aim investigations and treatments where they actually help [3,25,26]. However, despite extensive descriptive literature on alcohol-related seizures, there is limited quantitative data defining how often seizures in drinkers are truly withdrawal-related versus due to other causes, and no structured triage guidance validated in Indian emergency settings. This evidence gap formed the rationale for our study. In this study, we aimed to learn the demographics of patients presenting with alcohol-related seizures.

This article was originally presented as a thesis, titled "An Observational Study on Clinical Profile of Patients with Alcohol-Associated Seizures," to the National Board of Examinations, India, in 2021.

## Materials and methods

This was a hospital-based, single-centre, observational study undertaken at a tertiary care centre in India from January 2020 to February 2021. The Institutional Ethics Committee of Kasturba Hospital, Valsad, India, approved this study (Approval No. ECR/1273/Inst/GJ/2019). We prospectively enrolled 500 consecutive, homogenous adults who presented to the ED or acute medical unit with a seizure and had a history of alcohol use. For consistency of terminology and case classification, the team used the ICD-10 diagnostic framework for mental and behavioural disorders, and the DSM-5 criteria for alcohol use disorder and withdrawal states throughout the study period [5,6].

Participants and eligibility

Patients were eligible if they were ≥18 years and had a witnessed or strongly suspected epileptic seizure (convulsive or non-convulsive), documented by the treating team, together with self-reported, attendant-reported, or recorded evidence of alcohol use. We excluded presentations where an alternative, non-alcohol cause clearly applied at first contact (e.g., eclampsia), isolated psychogenic non-epileptic events, known epilepsy under regular follow-up where the current episode was judged unrelated to alcohol by the attending physician, and major head trauma requiring immediate neurosurgical triage. Enrolment was consecutive until the target of 500 patients was reached to minimize selection bias.

The objective of this study is to determine the proportion of alcohol-withdrawal-related versus non-withdrawal seizures among adults with alcohol use presenting to the ED and to identify bedside clinical and biochemical features distinguishing these groups and to describe short-term hospital outcomes.

Operational definitions

An AWS was defined as one or more GTCS occurring typically within 6-48 hours of the last alcohol intake, in the context of recent reduction or cessation, with or without other withdrawal features. Case classification was adjudicated a priori. Two physicians independently reviewed each presentation against prespecified criteria (hours since last drink; CIWA-Ar score; laboratory evidence of hypoglycaemia, ketoacidosis, or electrolyte disturbance; neuroimaging; and clinical documentation). Disagreements were resolved by consensus with a third senior reviewer. Reviewers were blinded to in-hospital outcomes.

This clinical picture reflects well-described pathophysiology, i.e., down-regulation of GABAergic inhibition, up-regulation of glutamatergic excitation, and increased network hyperexcitability, with the potential for kindling across repeated withdrawals [7-10]. A non-withdrawal, alcohol-associated seizure was applied when another proximate cause better explained the event in a person who drinks, including AKA, hypoglycaemia, significant electrolyte disturbance (e.g., hyponatraemia and hypomagnesaemia), structural brain disease, intoxication-related threshold lowering, or other acute medical precipitants [7-12]. Withdrawal severity was measured with the CIWA-Ar at the point of first assessment, and serially where clinically indicated [16].

Assessment pathway

All patients underwent a structured history and examination focused on time since last drink, typical quantity and pattern, prior withdrawal/AWS episodes, comorbid illness and medicines, and focal neurological signs. The CIWA-Ar was recorded by trained staff using the original description and scoring anchor points [16]. To capture common precipitants and to characterize possible AKA early, initial laboratory testing included capillary/venous glucose; venous or arterial blood gas, with calculation of the anion gap and ketone assessment; serum sodium, potassium, chloride, bicarbonate, calcium, and magnesium; and renal and hepatic panels. These measures reflect the ED profiles and biochemical patterns described in classic and contemporary reports of AKA and alcohol-related metabolic derangements [21-23,25].

Neuroimaging with non-contrast-enhanced CT head was obtained at presentation when the semiology was focal, the history or timing was atypical or uncertain, there was head injury or anticoagulation, or when there was delayed recovery of consciousness. MRI was pursued when CT was inconclusive but clinical concern for structural pathology persisted. EEG was not performed routinely in straightforward AWS; it was reserved for cases with focal features, recurrent events without a clear withdrawal context, prolonged altered awareness, or when epileptic comorbidity was suspected, consistent with prior clinical literature on yield and indications [10-12,26].

Treatment framework

When AWS was the leading working diagnosis, patients were managed with benzodiazepines as first-line therapy, using symptom-triggered dosing or front-loading at the clinician’s discretion, with close cardiorespiratory monitoring. Airway protection, IV fluids, and electrolyte repletion were provided as needed, and parenteral thiamine preceded carbohydrate administration in at-risk individuals. This approach aligns with ED evidence on preventing recurrent alcohol-related seizures and with guidance favouring risk-stratified, symptom-triggered care [17-20].

When metabolic precipitants were identified - particularly AKA or hypoglycaemia - management followed established emergency protocols: rapid dextrose (after thiamine when deficiency was possible), isotonic fluids, correction of electrolytes (including magnesium), and targeted treatment of anion-gap metabolic acidosis and ketonaemia, as described in classic and modern sources and contemporary clinical summaries [21-23,25]. Where structural, infectious, haemorrhagic, or other medical causes were uncovered, care proceeded along standard speciality pathways, with neurology or neurosurgery input as required.

The primary outcome was adjudicated aetiology of the presenting seizure, classified as withdrawal versus non-withdrawal (with non-withdrawal subcategorized into metabolic, structural, intoxication-related, and other medical causes). Secondary outcomes included the need for ICU or step-up care, in-hospital seizure recurrence, length of stay, and in-hospital and seven-day mortality. We also recorded whether EEG, when obtained, altered immediate management, anticipating limited utility in clear AWS, based on prior reports [10-12,18-20].

Data were recorded prospectively on standard case record forms at the point of care and cross-checked against the electronic health record. Key variables (time since last drink, CIWA-Ar score, glucose/ketone/anion-gap results, and core electrolytes) were entered for internal consistency. Staff involved in CIWA-Ar scoring received a brief refresher, with examples, before the study start, referencing the original description [16].

Study size and statistical analysis

The sample size of 500 consecutive cases was chosen to provide adequate statistical power and precision for the study objectives. Based on prior literature, we anticipated that approximately 40% of seizures in individuals with alcohol use would be alcohol-withdrawal-related [7,12]. With 500 participants, the 95% CI around this estimate would have a margin of error of about ±4%, ensuring precise descriptive estimates. This sample size also provided >80% power (at a two-sided α = 0.05) to detect an absolute difference of at least 10% in key predictors between groups (e.g., CIWA-Ar score, glucose derangements, or sodium abnormalities).

Continuous variables were summarized as means with standard deviations, or medians with interquartile ranges, as appropriate, while categorical variables were presented as counts with percentages (n, %). Comparisons between withdrawal and non-withdrawal groups were performed using χ² or Fisher’s exact tests for categorical data, and t-tests or Mann-Whitney U tests for continuous data, as appropriate. Independent predictors of AWS were examined using multivariable models informed by clinical plausibility and prior evidence (including time since last drink, CIWA-Ar score, daily alcohol quantity, history of prior withdrawal seizures, serum sodium, glucose/ketones, and presence of head injury) [7-20]. Analyses were performed using standard statistical software, and a two-sided p < 0.05 was considered statistically significant.

## Results

We enrolled 500 adults presenting with a seizure and any history of alcohol use. The overall mean age was 49.9 ± 11.1 years, and 458/500 (91.6%) were men. Drinking frequency in the overall cohort was: 305/500 (61.0%) daily or near-daily, 145/500 (29.0%) three to five days per week, and 50/500 (10.0%) two days per week or less. The primary beverage consumed was country liquor/local spirits in 258/500 (51.6%), commercial spirits in 180/500 (36.0%), and beer in 62/500 (12.4%). Tobacco use was recorded in 280/500 (56.0%), diabetes in 92/500 (18.4%), and high blood pressure in 115/500 (23.0%). On screening, 412/500 (82.4%) met criteria consistent with alcohol dependence.

Table 1 summarizes baseline demographics and drinking patterns by seizure aetiology. Compared with participants whose seizures were due to other causes (n = 310), participants with alcohol-withdrawal-related seizures (n = 190) had a higher proportion reporting daily or near-daily drinking (140/190, 73.7% vs. 165/310, 53.2%) and a higher proportion screening positive for dependence (172/190, 90.5% vs. 240/310, 77.4%). Primary beverage differed by group: country liquor/local spirits were more common in the withdrawal group (120/190, 63.2% vs. 138/310, 44.5%), while beer and commercial spirits were relatively more common in the non-withdrawal group. Significant between-group differences were observed for age (p = 0.013), daily or near-daily drinking (p < 0.001), dependence on screening (p < 0.001), primary beverage (country liquor, commercial spirits, beer; all p < 0.05), tobacco use (p = 0.039), and diabetes (p = 0.044). No significant difference was observed for high blood pressure (p = 0.115).

**Table 1:** Baseline demographics and drinking patterns by seizure aetiology Data are n (%). Age is presented as mean ± standard deviation. Comparisons between alcohol-withdrawal-related seizures (n = 190) and seizures due to other causes (n = 310) were performed using Student’s t-test for age and the Chi-square test for categorical variables. All tests were two-sided; p < 0.05 was considered statistically significant.

Variable	All (n = 500)	Withdrawal (n = 190)	Other (n = 310)
Age, years - mean (standard deviation)	49.8 (11.1)	48.3 (10.6)	50.8 (11.3)
Daily or near-daily drinking - count (percentage)	305 (61.0%)	140 (73.7%)	165 (53.2%)
Dependence on screening - count (percentage)	412 (82.4%)	172 (90.5%)	240 (77.4%)
Primary beverage: country liquor or local spirits - count (percentage)	258 (51.6%)	120 (63.2%)	138 (44.5%)
Primary beverage: commercial spirits - count (percentage)	180 (36.0%)	55 (28.9%)	125 (40.3%)
Primary beverage: beer - count (percentage)	62 (12.4%)	15 (7.9%)	47 (15.2%)
Tobacco use - count (percentage)	280 (56.0%)	118 (62.1%)	162 (52.3%)
Diabetes - count (percentage)	92 (18.4%)	26 (13.7%)	66 (21.3%)
High blood pressure - count (percentage)	115 (23.0%)	36 (18.9%)	79 (25.5%)

**Figure 1:**
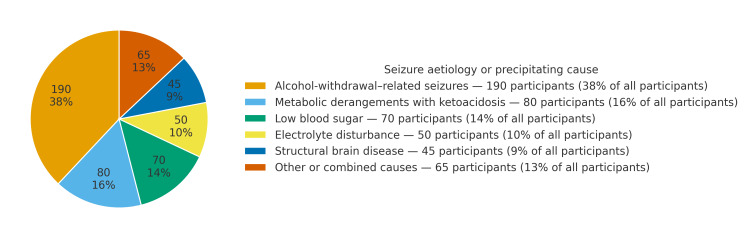
Seizure aetiologies in the cohort (n = 500) Values are n (%) of the overall cohort. Alcohol-withdrawal-related seizures (n = 190, 38.0%) and seizures due to other causes (n = 310, 62.0%) are shown. Non-withdrawal causes included metabolic derangements with ketoacidosis (80/500, 16.0%), hypoglycaemia (70/500, 14.0%), primary electrolyte disturbance (50/500, 10.0%), structural brain disease (45/500, 9.0%), and other or combined causes (65/500, 13.0%).

Aetiologies of the index seizure are summarized in Figure 1 and described below. By adjudication, 190/500 (38.0%) of seizures were classified as alcohol-withdrawal-related, and 310/500 (62.0%) were due to other causes. The distribution of non-withdrawal causes was: metabolic derangements with ketoacidosis, 80/500 (16.0%); low blood sugar, 70/500 (14.0%); primary electrolyte disturbance, 50/500 (10.0%); structural brain disease, 45/500 (9.0%); and other or combined causes, 65/500 (13.0%).

**Figure 2:**
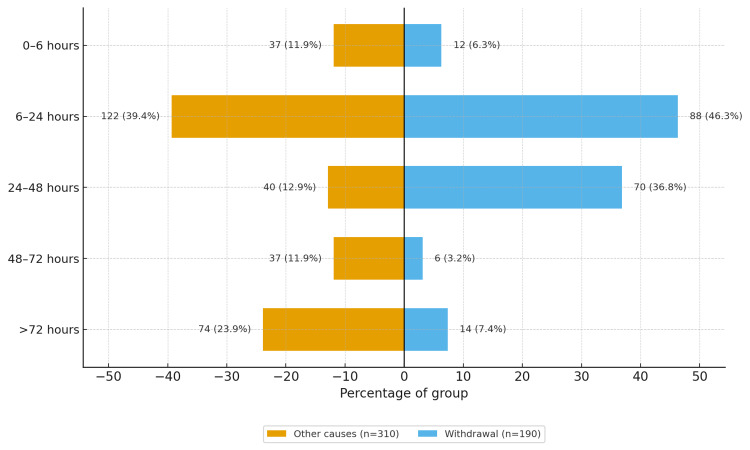
Hours since last alcohol drink at presentation by seizure aetiology Values are n (%) within each aetiologic group: alcohol-withdrawal-related seizures (n = 190) and seizures due to other causes (n = 310).

Time since last alcohol drink at presentation is shown in Figure 2. Alcohol-withdrawal-related seizures clustered in the early withdrawal windows (predominantly within the first 24-48 hours after last drink), whereas seizures due to other causes were more dispersed across categories and included a larger proportion presenting beyond 72 hours. Exact counts and percentages, by time window and by aetiologic group, are presented in Figure 2.

**Figure 3:**
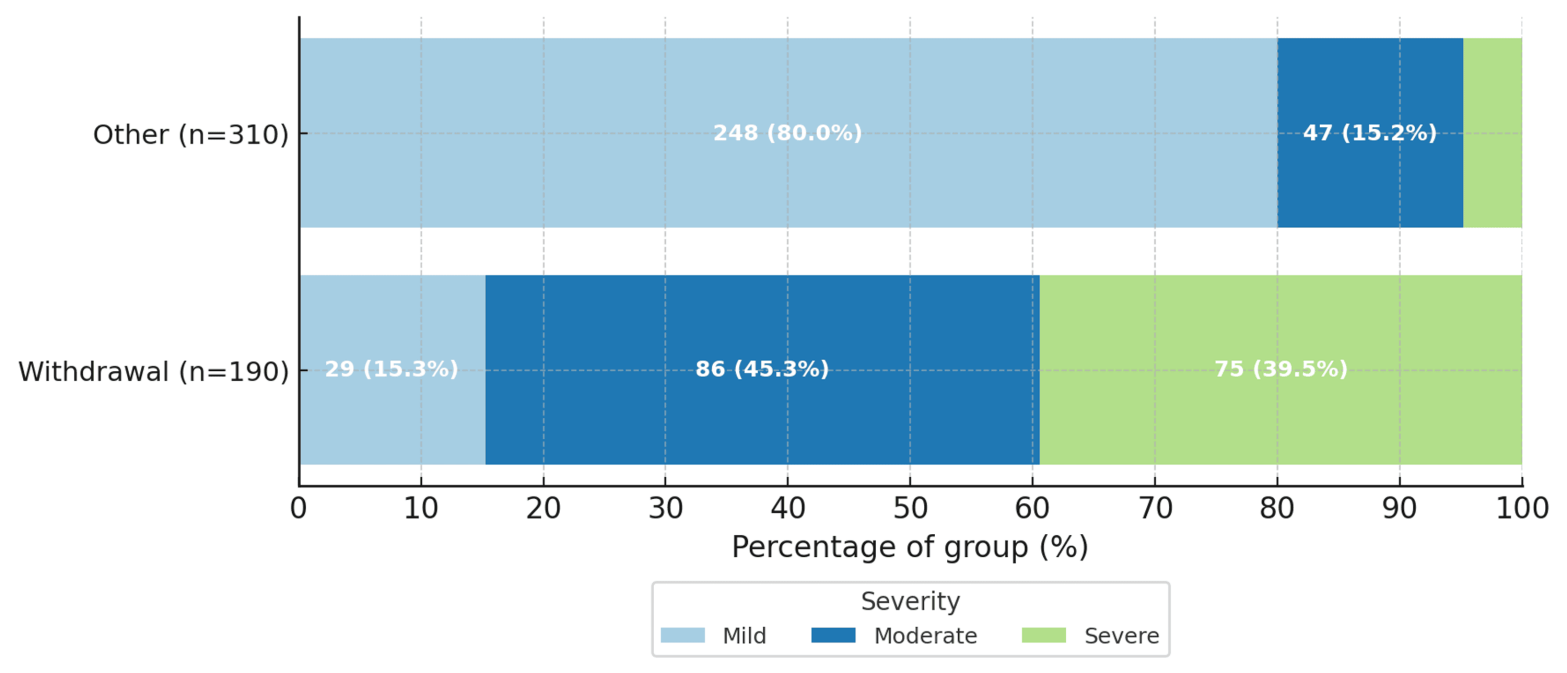
Withdrawal symptom severity at presentation by seizure aetiology as measured by the CIWA-Ar Severity categories are presented as n (%) within each aetiologic group: alcohol-withdrawal-related seizures (n = 190) and seizures due to other causes (n = 310). Severity cutoffs: mild CIWA-Ar <10, moderate 10-18, severe ≥19. CIWA-Ar, Clinical Institute Withdrawal Assessment for Alcohol-Revised

At triage, withdrawal severity was assessed using the CIWA-Ar. CIWA-Ar scores and categorical severity are summarized in Figure 3. A greater proportion of participants adjudicated as alcohol-withdrawal-related had moderate or severe CIWA-Ar scores than those with seizures due to other causes; CIWA-Ar numeric cutoffs for mild, moderate, and severe are provided in the Methods section.

Presenting vital signs and neurologic state at triage are summarized in Table 2. Median Glasgow Coma Scale (GCS) was 14 (interquartile range, 13-15) in both groups. Mean body temperature, oxygen saturation, and systolic blood pressure were broadly similar between groups. Seizure clustering at presentation (recurrent motor events within the same presentation) was more commonly recorded among the withdrawal group than the non-withdrawal group. Significant differences were observed for seizure clustering (p < 0.001), history of prior withdrawal (p < 0.001), heart rate (p < 0.001), systolic blood pressure (p < 0.001), body temperature (p < 0.001), and head injury at presentation (p = 0.034). No significant difference was observed for oxygen saturation (p = 1.000).

**Table 2:** Presentation and clinical state at Emergency Department triage by seizure aetiology Data are n (%) for categorical variables, mean ± standard deviation for normally distributed continuous variables, and median (interquartile range) for skewed variables. Comparisons between alcohol-withdrawal-related seizures (n = 190) and seizures due to other causes (n = 310) were performed using Student’s t-test for normally distributed continuous variables, Mann-Whitney U test for non-normally distributed continuous variables (CIWA-Ar and GCS), and Chi-square or Fisher’s exact test (if expected cell counts <5) for categorical variables. All tests were two-sided; p < 0.05 was considered statistically significant. CIWA-Ar, Clinical Institute Withdrawal Assessment for Alcohol-Revised; GCS, Glasgow Coma Scale

Variable	All (n = 500)	Withdrawal (n = 190)	Other (n = 310)
CIWA-Ar score at triage - median (interquartile range)	11 (6-16)	18 (14-23)	6 (3-9)
Seizure clustering at presentation - count (percentage)	140 (28.0)	85 (44.7)	55 (17.7)
History of prior withdrawal - count (percentage)	220 (44.0)	130 (68.4)	90 (29.0)
Heart rate, beats per minute - mean (standard deviation)	97.8 (18.3)	104 (20)	94 (16)
Systolic blood pressure, millimetres of mercury - mean (standard deviation)	131.8 (22.1)	138 (24)	128 (20)
Oxygen saturation by pulse oximetry, per cent - mean (standard deviation)	97 (3)	97 (3)	97 (3)
Body temperature, degrees Celsius - mean (standard deviation)	37.2 (0.6)	37.4 (0.6)	37.1 (0.6)
GCS at triage - median (interquartile range)	14 (13-15)	14 (13-15)	14 (13-15)
Head injury at presentation - count (percentage)	45 (9.0)	10 (5.3)	35 (11.3)

**Figure 4:**
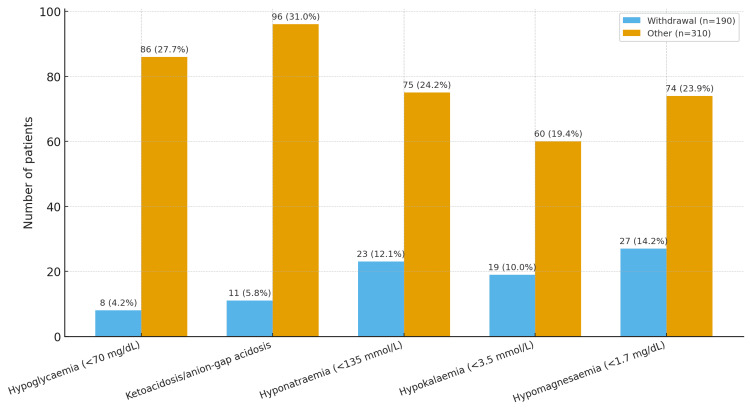
Selected laboratory abnormalities at presentation by seizure aetiology Values are n (%) within each aetiologic group: alcohol-withdrawal-related seizures (n = 190) and seizures due to other causes (n = 310). Laboratory thresholds: hypoglycaemia = blood glucose <70 mg/dL; ketoacidosis/anion-gap acidosis = positive serum or urine ketones with elevated anion gap; hyponatraemia = sodium <135 mmol/L; hypokalaemia = potassium <3.5 mmol/L; hypomagnesaemia = magnesium <1.7 mg/dL.

Laboratory abnormalities at presentation are shown by aetiologic group in Figure 4. Neuroimaging (non-contrast CT head) was performed in 325 (65.0%) participants, revealing structural abnormalities in 45 (9.0%), all belonging to the non-withdrawal group. Imaging was prioritized for patients with focal features, head injury, or delayed postictal recovery. Liver function tests were available for 472 (94.4%) participants, of whom 99 (21.0%) had mild to moderate transaminase elevation, 42 (9.0%) had elevated total bilirubin, and 28 (6.0%) had raised serum ammonia levels. These abnormalities were comparable between alcohol-withdrawal-related and non-withdrawal seizures and were not significantly associated with seizure aetiology. Ketone/anion-gap positivity was near-universal among participants whose seizures were attributable to metabolic ketoacidosis (metabolic subgroup, n = 80); hypoglycaemia (low blood sugar); hyponatraemia (low sodium); hypokalaemia (low potassium); and hypomagnesaemia (low magnesium).

Treatment and in-hospital course are summarized in Table 3. Benzodiazepines were administered to 280/500 (56.0%) overall, predominantly for clinically significant withdrawal. Electrolyte replacement and targeted management for metabolic derangements were given in the respective subgroups. EEG was performed in 220/500 (44.0%) of participants and was used selectively. Need for higher acuity or ICU-level care occurred in 60/500 (12.0%); in-hospital seizure recurrence occurred in 55/500 (11.0%); median length of stay was 4 (interquartile range, 2-6) days; and in-hospital death occurred in 12/500 (2.4%).

**Table 3:** In-hospital course and outcomes in the overall cohort Data are n (%) for categorical outcomes and median (interquartile range) for length of stay. Events are reported independently and are not mutually exclusive; totals do not sum to 500. All values are descriptive.

Outcome	Overall cohort (n = 500)
Need for higher-acuity or ICU-level care	60/500 (12.0%)
In-hospital seizure recurrence	55/500 (11.0%)
Length of stay, days	4 (2-6)
In-hospital death	12/500 (2.4%)

## Discussion

In this 500-patient emergency cohort with a history of alcohol use, nearly two-thirds of index seizures were attributable to causes other than alcohol withdrawal, with metabolic derangements - especially AKA and low blood sugar - being the most prominent among alternative explanations. This distribution emphasizes that, while alcohol-withdrawal-related seizures remain common, the default assumption that “seizure in a drinker = withdrawal” is unsafe. Our comparative data show a coherent clinical pattern that helps separate the two pathways at the bedside: alcohol-withdrawal-related events clustered tightly within 6-48 hours of the last drink and presented with higher withdrawal symptom burden, more seizure clustering, and greater autonomic activation, whereas seizures due to other causes were temporally more dispersed and metabolically abnormal far more often (Tables 1-2; Figures 2-4).

The timing signal aligns with long-standing neurobiology and clinical descriptions of alcohol withdrawal: down-regulated GABAergic inhibition, up-regulated glutamatergic excitation, and network hyperexcitability with kindling across repeated withdrawals produce the familiar window in which GTCS are most likely to emerge [7-15]. In our series, 46.3% of withdrawal cases presented 6-24 hours from last drink, and 36.8% presented 24-48 hours, whereas the “other-causes” group showed a heavier right tail beyond 48 hours, including 17.7% arriving >72 hours after last intake (Figure 2). These differences, coupled with the higher CIWA-Ar scores at triage in the withdrawal group (median 18 vs. 6), make a strong case for embedding formal withdrawal scoring, rather than impressionistic labels, into first-hour triage [16].

The metabolic signature in seizures due to other causes was equally instructive. Low blood sugar, ketones or anion-gap acidosis, and abnormalities of blood sodium, potassium, and magnesium were each two to five times more frequent in the non-withdrawal group (Figure 4). This pattern mirrors classic and contemporary reports of AKA and hypoglycaemia in people who drink, where rapid glucose, fluids, thiamine before carbohydrates, and aggressive electrolyte repletion are the time-critical steps [21-23,25]. Given the overlap between heavy alcohol use, malnutrition, and thiamine deficiency, early parenteral thiamine remains a low-risk, high-yield intervention to reduce the risk of Wernicke-Korsakoff syndromes [24]. Our practice pathway and selective use of EEG and neuroimaging also track prior guidance: EEG rarely alters immediate management in straightforward withdrawal; imaging should be directed by focal features, atypical timing, head injury, or delayed recovery of consciousness [10-12,26].

Structural brain lesions represented a smaller but clinically important subset of seizure aetiologies in our cohort. All structural causes were confirmed radiologically, most often chronic post-stroke gliosis, traumatic sequelae, or intracranial space-occupying lesions detected on non-contrast CT. These findings are consistent with prior reports showing that 5%-15% of alcohol-associated seizures may arise from coexisting structural abnormalities rather than withdrawal alone [10,11]. Although CT scanning is rapid and widely available, universal imaging for every GTCS is neither practical nor routinely recommended. International emergency medicine and neurology guidelines endorse selective imaging based on focal features, head injury, atypical timing, or delayed postictal recovery, to optimize diagnostic yield while minimizing unnecessary radiation and cost [27].

Baseline differences provide clinical nuance. Individuals with alcohol-withdrawal-related seizures were younger, more often daily or near-daily drinkers, and screened more frequently as dependent, while diabetes and high blood pressure were common in seizures due to other causes (Table 1). These contrasts are congruent with literature linking heavier, longer-standing consumption to withdrawal syndromes on the one hand, and cardiometabolic comorbidity - and therefore metabolic precipitants - on the other [1-4,7-13]. The predominance of country liquor/local spirits in the withdrawal group also reflects local drinking patterns, reminding clinicians that context matters for pre-test probability and for counselling after the acute event (Table 1).

The in-hospital course in our cohort was short but not trivial. Roughly one in eight patients required step-up or intensive care, and one in nine had seizure recurrence, typically within 24 hours; in-hospital mortality was 2.4% (Table 3). These outcomes sit within ranges reported in emergency and acute medicine series and underscore the dual mandate of early risk stratification and reversible-cause hunting [9,17-20]. Critically, the data suggest a pragmatic, reproducible triage sequence: anchor on time since last drink and a scored withdrawal assessment; check bedside glucose immediately; obtain electrolytes (including magnesium), and acid-base/ketones where available; and escalate to neuroimaging when semiology is focal, timing is atypical, head injury is present, or recovery is delayed.

This study has several limitations, as with any pragmatic emergency case series. Despite prespecified criteria and structured review, some misclassification is likely when withdrawal coexists with metabolic derangement or structural disease; such overlap is not rare in clinical practice [7-13,21-23]. Laboratory panels and timing were not identical in every case, which could inflate or depress the apparent prevalence of specific abnormalities. Nevertheless, the direction and relative magnitude of differences between groups were consistent across domains (Tables 1-2; Figure 4). The study depends on the time-since-alcohol-consumption history provided by patients, introducing possible recall bias. It was conducted at a single tertiary centre and therefore reflects local case mix, drinking patterns, and referral pathways - limiting generalizability to other regions and highlighting the need for multi-centre studies in diverse EDs. Finally, longer-term outcomes such as recurrent seizures, unplanned re-attendance, and engagement with alcohol-treatment services were not captured, restricting our findings to the acute inpatient phase.

Clinically, the message is practical. Time since last drink and structured withdrawal scoring sharpen diagnostic thinking but must be paired with a low threshold for identifying metabolic precipitants that have immediate, reversible treatments. When alcohol withdrawal is likely, benzodiazepines remain first-line; when metabolic triggers are suspected or confirmed, glucose, fluids, and electrolyte/thiamine protocols should proceed without delay - even while anticonvulsant and airway decisions are made [17-25]. A selective strategy for EEG and imaging avoids both under- and overuse, reserving these tests for the phenotypes most likely to benefit [10-12,26]. In settings where country liquor and heavy daily intake are prevalent, brief intervention and robust linkage to treatment should be standard parts of the post-acute plan, given the high proportion of screening consistent with dependence in this cohort (Table 1) and the wider public health burden of harmful alcohol use [1-4].

## Conclusions

In adults with a history of alcohol use who present to the ED with a seizure, most events are not due to alcohol withdrawal; rather, metabolic precipitants - especially ketoacidosis, low blood sugar, and abnormalities of blood sodium, potassium, and magnesium - are common and immediately treatable. The combination of time since last drink, a structured withdrawal assessment (e.g., the CIWA-Ar), and rapid bedside testing for glucose and electrolytes reliably separates AWS from seizures due to other causes and directs early, targeted therapy. Although the overall hospital stay was short and mortality low, the need for higher-acuity care and the frequency of early recurrence underscore the importance of systematic triage and prompt correction of reversible abnormalities. Embedding this simple approach into emergency pathways, paired with brief intervention, thiamine, and linkage to alcohol-treatment services, offers a route to safer care while also reducing recurrence and re-admission.
